# Dust radiative forcing and its impact on surface energy budget over West Africa

**DOI:** 10.1038/s41598-020-69223-4

**Published:** 2020-07-22

**Authors:** Abdoul Aziz Saidou Chaibou, Xiaoyan Ma, Tong Sha

**Affiliations:** Key Laboratory of Meteorological Disaster, Ministry of Education (KLME), International Joint Laboratory on Climate and Environment Change (ILCEC), Key Laboratory for Aerosol-Cloud-Precipitation of China Meteorological Administration, School of Atmospheric Physics, Collaborative Innovation Centre on Forecast and Evaluation of Meteorological Disasters, Nanjing University of Information Science and Technology, Nanjing, 210044 Jiangsu China

**Keywords:** Climate sciences, Planetary science

## Abstract

Dust is the dominant aerosol type over West Africa (WA), and therefore accurate simulation of dust impact is critical for better prediction of weather and climate change. The dust radiative forcing (DRF) is estimated using two sets of experiments in this study: one without and the other with dust aerosol and its feedbacks with the Weather Research and Forecasting with Chemistry model (WRF-Chem). Results show that DRF presents a net warming effect at the top-of-atmosphere (TOA) and in the atmosphere (ATM), and cooling at the surface (SFC). The net DRF over WA is estimated to be 9 W/m^2^ at the TOA, 23 W/m^2^ in the ATM, and − 13 W/m^2^ at the SFC. Furthermore, dust-induced a reduction of sensible heat up to 24 W/m^2^ and SFC temperature up to 2 °C cooling over WA, an increase of latent heat up to 12 W/m^2^ over Sahara, a decrease up to 24 W/m^2^ over the vegetated surfaces and an increase in the surface energy balance up to 12 W/m^2^ over WA. The presence of dust significantly influences the surface energy budget over WA, suggesting that dust effects should be considered in more climate studies to improve the accuracy of climate predictions.

## Introduction

Aerosols play a vital role in the climate system and have been among the major uncertainties in predictions of future climate change^1^. West Africa (WA) is one of the most vulnerable regions to climate change, with the Sahara desert as the major source of dust aerosols in the world^2–5^. The vulnerability is higher in the Sahel region, which has experienced a long period of drought in the late 1960s and 1980s. Numerous studies have pointed out that dust loading over the Sahel has increased significantly between the 1960s and 1980s, and is the consequence of drying of the region^6–13^. Since the 1990s, better rainfall conditions appear to occur in the Sahel region^14–16^. On this basis, a comprehensive investigation of dust impact on climate variability and drought in WA is essential, where the economy depends mostly on rainfed agriculture and transhumant livestock^11,15,17–19^. To improve our understanding, the scientific community has launched several field campaigns, such as SaHAran Dust Experiments (SHADE)^20^, SAharan Mineral dUst experiMent (SAMUM)^21^, African Monsoon Multidisciplinary Analysis (AMMA)^22^, etc.

Dust aerosols emitted from the Sahara and Sahel are considerably higher than any other desert in the world. The surface wind speed is the primary controlling factor of the emission and transport of dust particles into the atmosphere to great distances by convective events that develop actively in the desert^2,3,5,23,24^. More than half of dust deposited in the oceans comes from elsewhere in North Africa. Saharan dust contains nutrients that fertilize soils and water, block or reflect sunlight, affect the formation of clouds and cyclones^25–27^. Interactions of dust particles with radiation in the troposphere (absorption, scattering, etc.) are the basis in changing atmospheric state parameters, which may induce significant changes in climate. Dust aerosols influence many processes that modulate regional climate. Firstly, they exert a direct effect either through scattering or absorption of solar radiation that leads to a warming or cooling of the atmospheric layers in the case of absorption or reflection, respectively^28^. Secondly, the absorbing particles via a semi-direct effect inhibit cloud formation by diminishing the adiabatic cooling of the atmosphere because absorbing particles heat the cloud layer and cause cloud evaporation^29,30^. Finally, dust particles may cause indirect effects by altering cloud microphysics and precipitation acting as cloud condensation nuclei^31,32^. Therefore, to adequately predict dust impact on weather and climate radiative heating and cooling effects, these particles must be considered since dust aerosols alter the dynamics and thermodynamics of the atmosphere^33^.

Saharan dust storms have often been observed from space by remote sensing. Still, the total impact on the Earth's radiative budget has been challenging to assess due to the limited number of observations made from the surface, mainly in WA^34–36^. Improved assessment of dust impact on climate requires continuous observations from both satellites, and ground-based instrument networks^37,38^. However, satellite observations and ground-based measurements alone would not be sufficient to fully describe the spatiotemporal variability, heterogeneity, and different spectral behavior of dust aerosols. Therefore, the use of climate models becomes crucial to improve our understanding of dust distribution and its physical, chemical, and optical properties. Also, climate models have been used to study the effect of dust on radiation budgets^39–51^. Because of their sensitivity to different forcing, climate models still struggle in replicating observations^52–54^.

Given that, it is essential to continue improving the reliability of climate models to allow for a better assessment of dust impact on climate. Due to the very high dust burden frequently occurring during the year, WA is an ideal region to investigate the radiative forcing of aerosols^55,56^. This study uses the WRF-Chem model to examine the impact of dust aerosol on the radiation and surface energy budget over WA.

## Materials and methods

### Model description and experiments

**Figure 1:**
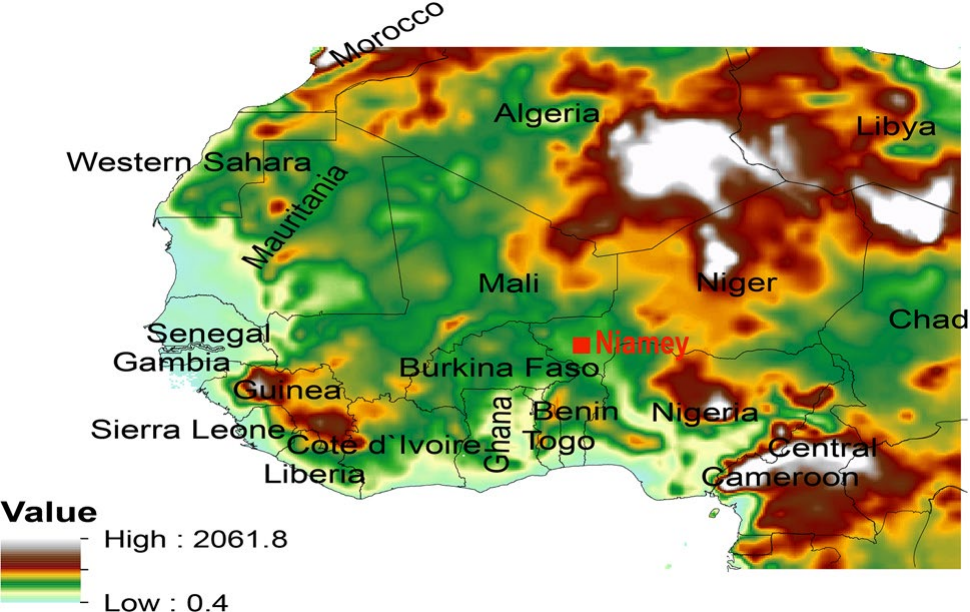
Model domain with the location of countries included in the study area superimposed on the surface elevation topography (m). The Atmospheric Radiation Measurements (ARM) mobile facility observation site in Niamey (2° E, 13° N) is indicated in red color.

We used version 4.0.2 of the Weather Research and Forecasting Model coupled with chemistry (WRF-Chem) to simulate dust impact on the radiation fluxes over the model domain presented in Fig. 1. WRF-Chem has been used in several studies to simulate processes such as the emission, transportation, deposition, vertical mixing, and chemical transformation of trace gases, aerosol interactions, photolysis, and radiation with meteorology^51,55–63^. In this study, WRF-Chem implements the Goddard Global Ozone Chemistry Aerosol Radiation and Transport (GOCART) dust scheme, which includes the emission, advection, and deposition^64,65^. The GOCART dust emission scheme is run in its default configuration in the version of the WRF-Chem model used in the present study. The simulation domain (0°–30° N and 20° W–20° E) covers WA at a resolution of 30 km and 51 vertical levels. The meteorological data used for initial and lateral boundary conditions was re-analysis data from the National Environmental Prediction Center (NCEP) available every 6 h at a spatial resolution of 1° × 1°.

The main physical and chemical parameterizations are summarized in Table 1. The microphysical scheme^66^ that includes ice, snow and graupel processes, was used for cloud physics. The Rapid Radiative Transfer Model for General Circulation Models (RRTMG) scheme^67^ used for both Longwave (LW) and Shortwave (SW) radiations includes the Monte Carlo Independent Column Approximation (MCICA) method of random cloud overlap^68^. The planetary boundary layer is chosen by the non-local K scheme of Yonsei University with an explicit training layer and a parabolic K profile in an unstable mixing layer. This scheme includes a topographic correction for surface winds to represent additional drag due to sub-grid topography and increased flow at the top of a hill^69^ and an option for descending mixture driven by radiative cooling. The cumulus parameter setting option^70,71^ chosen is Grell 3D, an improved version of the GD scheme. Noah's land surface model with four-layer temperature and soil moisture, split snow cover, and frozen soil physics was selected^72^. Finally, the Monin–Obukhov-based MM5 similarity scheme with the Carslon-Boland viscous underlayer and the standard similarity functions from the look-up tables was used^73–77^.

**Table 1:** Physical and chemical modeling options used.

Microphysics	Lin et al. (1983) scheme
Longwave radiation	Rapid radiative transfer model (RRTMG)
Shortwave radiation	Rapid radiative transfer model (RRTMG)
Surface layer	MM5 similarity scheme
Land surface	Unified Noah land surface model
Planetary boundary layer	Yonsei University scheme
Cumulus parameterization	Grell 3D Ensemble scheme
Meteorology initial and boundary conditions	National Environmental Prediction Center (NCEP)
Horizontal resolution	30 (km)
Vertical levels	51
Time step	Adaptive time stepping always below 180 s
Chemistry	Goddard Global Ozone Chemistry Aerosol Radiation and Transport (GOCART) simple aerosol scheme
Dust Emission	Include GOCART

In most desert areas, dust comes from sediments and alluvial deposits found in depressions, sedimentary basins, and ancient valleys. In the WRF-Chem model, dust source function is represented by the availability of loose erodible soil material^78,79^, and dust emission is computed as a function of wind energy, soil moisture, and particle size. A similar empirical formula developed^80^ is used to calculate the dust emission flux F in the model following expression:

1 $${\text{F}} = \left\{ {\begin{array}{*{20}l} {{\text{CSs}}_{{\text{p}}} {\text{u}}_{{10{\text{m}}}}^{2} \left( {{\text{u}}_{{10{\text{m}}}} - {\text{u}}_{{\text{t}}} } \right),} \hfill & { {\text{if u}}_{{10{\text{m}}}} > {\text{u}}_{{\text{t}}} } \hfill \\ {0,} \hfill & {{\text{otherwise}}} \hfill \\ \end{array} } \right.$$

where C is a dimensional factor equal to 1 µgs^2^ m^−5^, S is the source function^64^. u_10m_ is the horizontal wind speed at 10 m, u_t_ is the threshold 10 m wind velocity for initiating erosion, and s_p_ is the mass fraction of each particle size group which are between 0.1 and 6 μm. The threshold velocity for dust production is the most important parameter of the formula due to its dependence on the effects of vegetation residue, soil roughness, soil texture, and the effect of atmospheric precipitation^80^.

Dust particles in the model are distributed into five discrete size bins with an effective radius of 0.5, 1.4, 2.4, 4.5, and 8.0 μm. The emission within each bin is injected to the lowest model level, the chemical module computes the dispersion, and separate schemes estimate dust mass concentrations for transport and removal from the atmosphere^78^. The optical properties are computed as wavelength-dependent at four wavelengths (300, 400, 600, 999 nm) for shortwave radiation and 16 wavelengths for longwave radiation using Mie theory^81^ (i.e., the aerosol optical depth (AOD), the single scattering albedo (SSA), the asymmetry parameter (g)). The Mie theory assumes dust particles as perfect spheres and internally mixed in each size bins. This spherical approximation may result in model bias^82–84^ and inaccurate estimation of the scattering phase function for dust particles, important for remote sensing applications, however, the impact is insignificant on the radiative flux divergence that represents the climate forcing^39,85^. The dust refractive index is considered to be wavelength-dependent for SW and LW spectral bands. The refractive indices are calculated by volume averaging for each aerosol size bin. Angstrom exponent relationship could be used to convert wavelengths needed between the wavelengths mentioned above. Though the model outputs the extinction coefficient at 550 nm, and here AOD is calculated by integrating the extinction coefficient over the whole atmospheric column using Eq. 2:

2 $$AOD=\sum_{i=0}^{nlev}{EXT}_{i}*{D}_{{z}_{i}}=\sum_{i=0}^{nlev}{AOD}_{i}$$

where EXT_i_ is the extinction of the layer, and D_zi_ is the path of the level layer.

We ran simulations between May 22, 2006, and August 31, 2006, with the first ten days as a spin-up time that is not included in the analysis, and an adaptive time-stepping always below 180 s was used for numerical stability. The study period was chosen for the reason that Saharan aerosols predominate during summer months when dust events are associated with intense convective events such as squall lines that develop over WA^3,86,87^. We performed two sets of experiments, one without and the other with dust aerosol and its feedbacks. The first set of the experiment does not include dust aerosol and is considered as the control run (CTL) in which the chemistry was set to zero. The second set of the experiment (dust simulation) simulates the dust aerosol, and dust radiation feedback is turned on to consider the semi-direct effect (e.g., changes in clouds induced by radiative forcing). The difference between dust and CTL experiment allows us to examine the dust impact on radiation. Other parameterizations consist of convective transport of aerosols based on the Grell convection scheme^70,71^, vertical turbulent mixing based on non-local boundary layer vertical diffusion of Yonsei University^69^, and dry deposition from gravitational settling and surface deposition^88^. The sea surface temperature was updated in the simulations using data downloaded from NOAA/NCEP server (ftp://polar.ncep.noaa.gov/pub/history/sst/). Biomass burning emissions and indirect effects of dust were not taken into account in the simulation. However, indirect effects can be considered to be as significant as the direct effect and will be addressed in our future investigations. In all simulations, the same physical parameterizations were used. The effects of unresolved clouds are included in SW and photolysis schemes.

### Determination of radiation fluxes

The direct radiative forcing of aerosols is defined as the change in radiative flux due to its scattering and absorption by aerosols (including any feedbacks and natural processes)^33,50,89^. In general, the radiative forcing is estimated at the surface (SFC), at the top-of-atmosphere (TOA) and in the atmosphere (ATM). In the present work, the radiative forcing of dust (DRF) is calculated by the difference between the net (downward minus upward) radiative fluxes (irradiance) with and without dust in the model over the study area, according to the following formula^33,90,91^:

3 $$\Delta F=\left({F}_{DUST}^{\downarrow }-{F}_{CTL}^{\downarrow }\right)-\left({F}_{DUST}^{\uparrow }-{F}_{CTL}^{\uparrow }\right)$$

where ΔF (W/m^2^) is the net radiation, F_DUST_ and F_CTL_ are irradiances with and without dust in the model, ↓ ↑ indicates the direction of the irradiances downwards and upwards, respectively.Different surface energy flows provided by the model are used to calculate total surface fluxes over the study area. The energy balance at the surface is more complicated than that at the TOA because the energy flows by conduction and convection of heat and moisture through turbulent fluid motion must be considered in addition to the radiation^92^. The surface energy balance is composed of four main terms: net radiation, sensible heat flux, latent heat flux, and ground heat flux, given here by the surface energy budget (Q) equation^93,94^:

4 $$Q={LW}^{\downarrow }-{LW}^{\uparrow }+\left(1-A\right)*{SW}^{\downarrow }-(SH+LH+GH)$$

Q is positive as a gain of energy and negative when loss. LW and SW are longwave and shortwave radiation fluxes. A (surface albedo) is a fraction of the incoming solar radiation reflected upward from the Earth's surface. GH (Ground Heat flux) is the energy loss through the lower boundary by heat conduction^92,93^. SH (Sensible Heat flux) and LH (Latent Heat flux) are positive upward, and represent the energy loss from the Earth’s surface to the atmosphere associated with heat transfer and evaporation, respectively^94^. All these radiation fluxes are in W/m^2^ and positive quantities^93^. The estimation of dust impact is obtained in terms of anomalies, defined for surface energy fluxes and temperature, by the difference between the dust and CTL simulation.

### Observational data

Before investigating dust impact on radiation, the model's ability to reproduce the spatial and temporal patterns were assessed with several sources of datasets over the study region. Toward this purpose, we combined data from the Multi-angle Imaging SpectroRadiometer (MISR), Atmospheric Radiation Measurements (ARM) mobile facility field campaign in Niamey Niger, and the Clouds and the Earth's Radiant Energy System (CERES) to assess the model outputs.

The first data used is AOD from the MISR instrument onboard the NASA Terra platform. MISR was launched in 2000 and observes continuously at nine distinct zenith angles, ranging from 70° afterward to 70° forward, and in four spectral bands 446, 558, 672 and 866 nm (blue, green, red, and near-infrared) and nine cameras at different angles. The Angstrom relationship is used to calculate AOD and SSA at 550 nm between 446 and 558 nm, for conformity with model results. The unique blend of directional and spectral data of MISR allows aerosol retrieval algorithms that do not depend on explicit radiometric surface properties to be used^95^. As such, MISR can retrieve aerosol properties over a variety of terrain, including bright surfaces like deserts^95,96^.

The second data used are AOD, SSA, surface radiation fluxes and temperature from the ARM mobile facility field campaign in Niamey Niger in 2006, funded by the U.S. Department of Energy's Office of Science, to provide key information for the AMMA project. The ARM mobile facility stationed at the Niamey airport (2°E, 13°N) and is equipped with fully active and passive instruments provides a wide range of atmospheric measurements^97^. The AOD is available at seven wavelengths 340, 380, 440, 500, 675, 870, 1,020 nm, and SSA at three wavelengths 467, 550, 660 nm. Here, AOD level 1 at 550 nm is also calculated using the Angstrom exponent between 500 and 675 nm, for consistency with the modeled one. The surface radiative flux measurements are SW and LW downwelling and upwelling components from broadband radiometers. Other parameters are temperature, SH, and LH measurements of the turbulent fluxes made using an eddy correlation measurement technique.

The last of the data used are measurements from the CERES instruments. CERES is a National Aeronautics and Space Administration (NASA) satellite project dedicated to observing the Earth's TOA global energy budget and estimate SFC and within ATM radiation budgets^98^. CERES instruments fly on the Terra, Aqua, Suomi National Polar-Orbiting Partnership (SNPP), and NOAA-20 satellites. Terra is in a descending sun-synchronous orbit with an equator-crossing time of 10:30 local time, while Aqua, SNPP, and NOAA-20 are in ascending sun-synchronous orbits with a 13:30 local time equator-crossing time^99,100^. Each CERES instrument measures filtered radiances in the SW (between 0.3 and 5 µm), total (between 0.3 and 200 µm), and window (between 8 and 12 µm) regions (CERES on NOAA-20 replaces the window channel with a LW channel)^99,100^. The CERES_EBAF_Ed4.1 product monthly and climatological averages of observed TOA and computed SFC all-sky fluxes are used in this study.

## Results and discussion

### Aerosol optical properties

**Figure 2:**
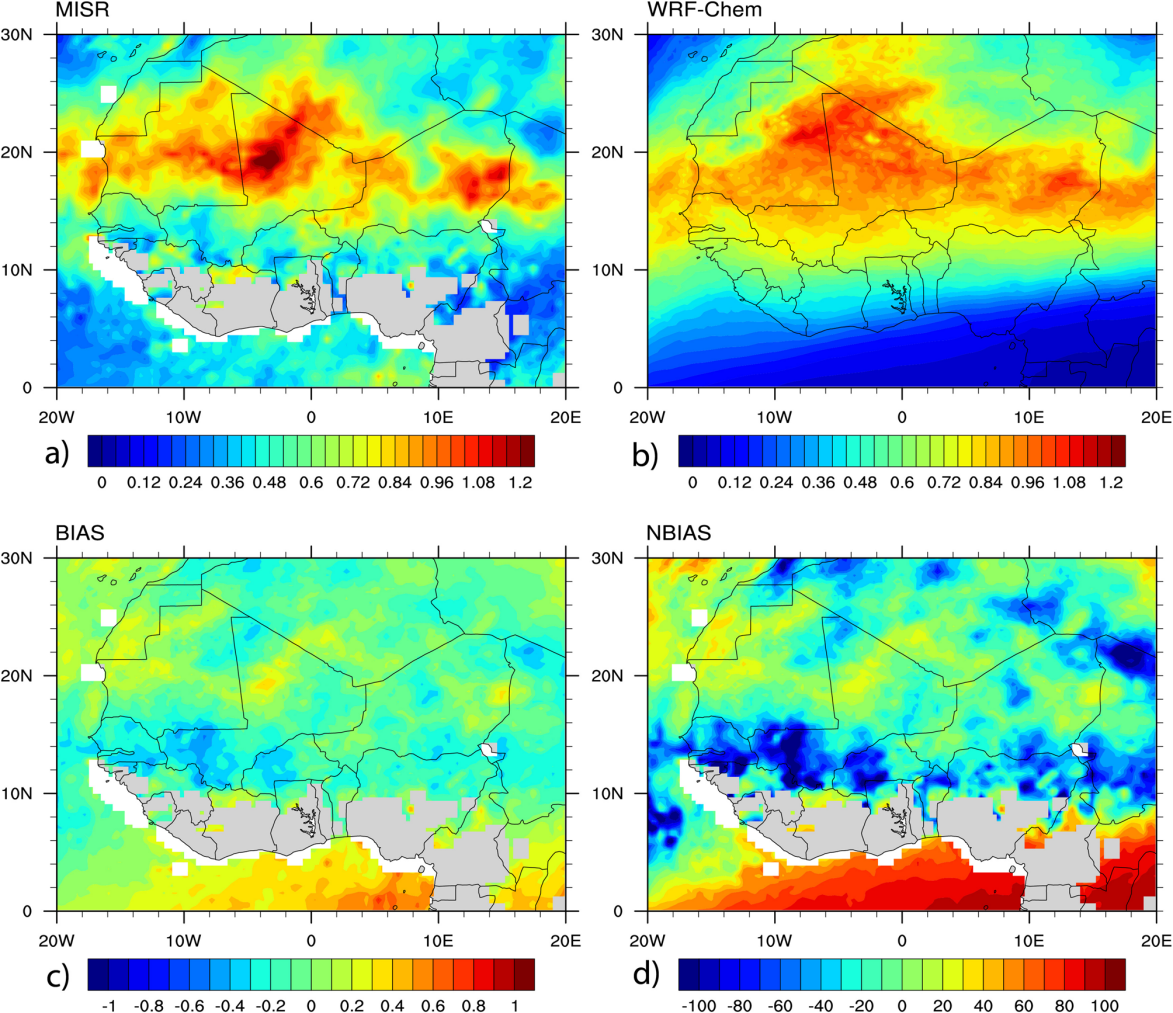
Spatial distribution of AOD averaged over June–July–August 2006 at 550 nm. Results are shown for: (**a**) MISR, (**b**) WRF-Chem, (**c**) bias between MISR and WRF-Chem, (**d**) normalized bias between MISR and WRF-Chem in %. The model AOD is sampled at the same overpass time as Terra (10:30). The gray-blank area in the plots is due to missing values.

AOD is a commonly used parameter to assess model outputs with observations. It is also available as a satellite standard product, and a frequently measured variable in the field experiments^101^. Figure 2 shows the spatial distribution of AOD at 550 nm derived from MISR (Fig. 2a) and WRF-Chem (Fig. 2b), and their bias (Fig. 2c) and normalized bias (Fig. 2d) averaged over June–July–August 2006. The model results are sampled at the same overpass time as Terra for comparison purposes. The MISR and model present a similar spatial distribution of AOD. Maximum values from MISR are observed over the Saharan Heat Low (SHL) region (Mauritania, Mali and Algeria, 12° W to 2° E, 15° to 27° N), which plays a vital role in the WA monsoon system^102–105^. A higher peak is also observed in the lee of Aïr and Adrar Mountains in Niger (2° to 10° E, 16° to 24° N) and the Bodélé Depression (border of Niger-Chad, 14° to 24° E, 14° to 20° N), which is the main source of dust in northern Lake Chad^106^. The model shows the same areas of maximum AOD observed from MISR. However, it over predicts these values mainly in the southern part of WA below 10° N, indicated by the calculated bias and normalized bias. This could be attributed to the model settings, such as the calculation of the threshold wind speed in the scheme, dust emission scheme, model configuration, lateral meteorological conditions, and surface properties. Our experiment missed the indirect effects of dust, which could also affect the simulated results in replicating the observation and radiative properties. The model is in good agreement with MISR in reproducing the spatial distribution of AOD.

**Figure 3:**
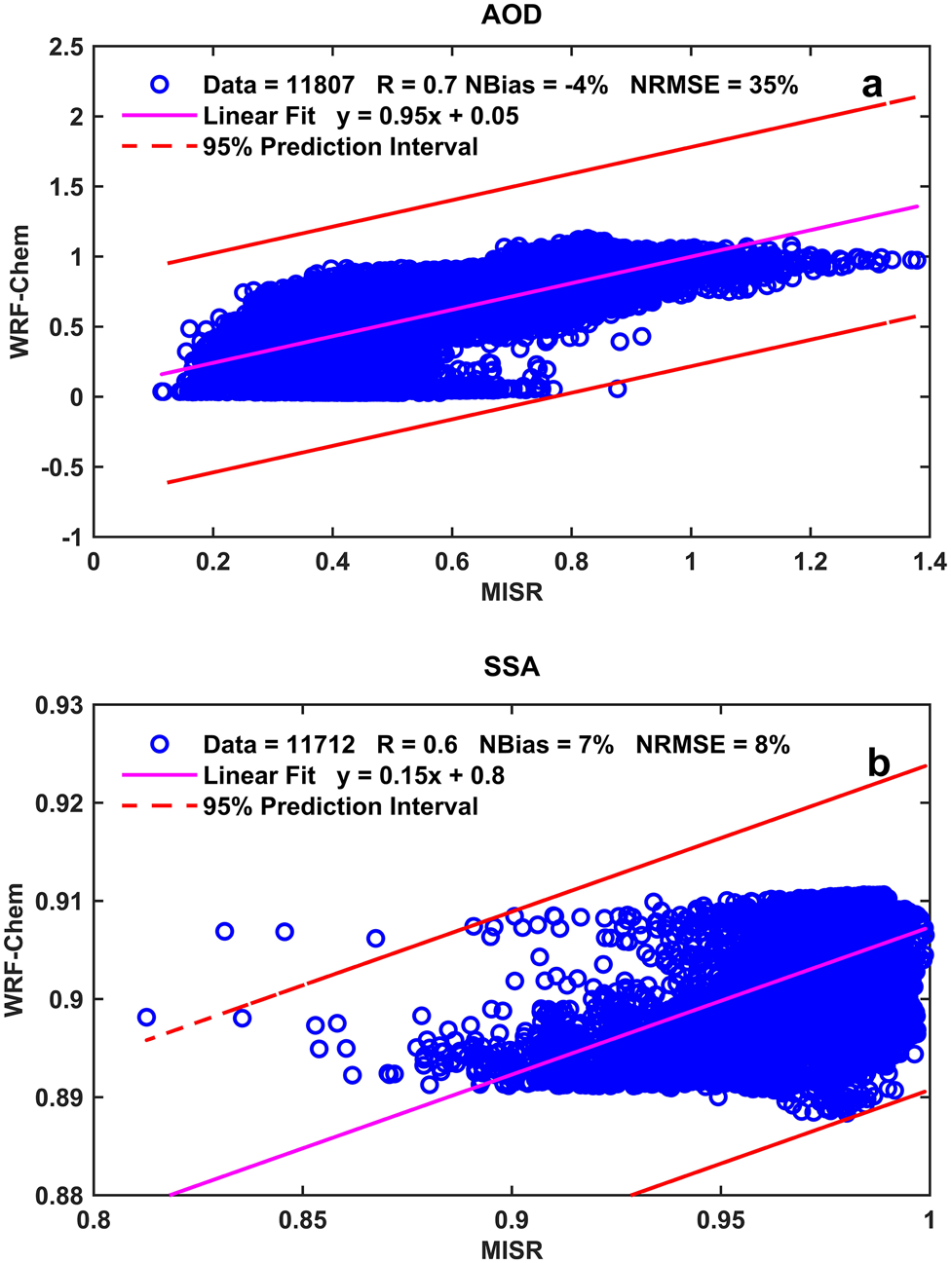
Agreement between MISR observation and WRF-Chem simulation. The plot shows the number of matched data points (circles), the regression line (solid magenta lines), and the error bounds (dashed red lines) of the regression, correlation coefficients, normalized bias, and root mean square error. Results are shown for: (**a**) AOD and (**b**) SSA, pixel to pixel comparison over WA.

**Figure 4:**
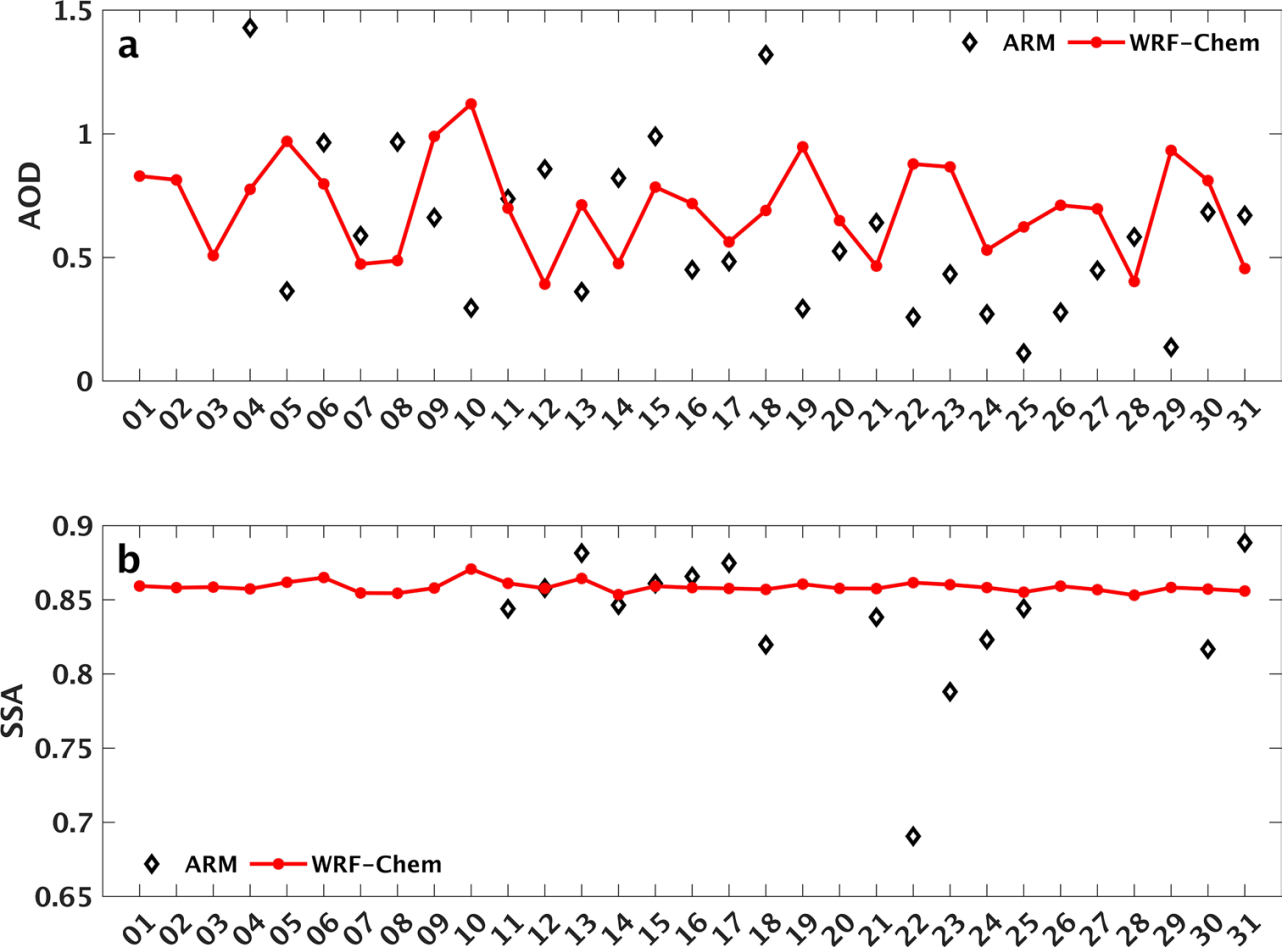
Daily AOD and SSA during August 2006 at 550 nm from ARM Facility Mobile observation at the Niamey, and WRF-Chem simulation. Results are shown for: (**a**) AOD from ARM in black polygon square and WRF-Chem in red line, (**b**) SSA from ARM in black polygon square, and WRF-Chem in red line. The ARM station in Niamey is indicated in Fig. 1 in red color.

Figure 3 presents the linear least squares regression analysis results of AOD and SSA at 550 nm between MISR and WRF-Chem simulation over the study area using a 95% prediction interval pixel to pixel comparison of the two datasets. Generally, the two datasets agree reasonably for both AOD (Fig. 3a) and SSA (Fig. 3b). Correlation coefficients of 0.7 and 0.6 were reported between the two datasets for both AOD and SSA, respectively, indicating a positive linear relationship. The normalized bias and root mean square error noted a satisfactory agreement between MISR and WRF-Chem. A normalized bias and normalized root mean square error of − 4% and 35% were calculated between the two datasets for AOD. For the SSA, the two datasets recorded the value of 7% for the normalized bias, and 8% for the normalized root mean square error. Figure 4 shows the temporal distribution of AOD and SSA at 550 nm from ARM and WRF-Chem simulation at the Niamey station. The ARM station in Niamey is shown in Fig. 1 in red color. The MISR AOD and SSA are not shown due to its inadequate temporal coverage at the Niamey station. The ARM AOD and SSA were not available for June and July 2006, the month of August 2006 is plotted here. The ARM and WRF-Chem show similar temporal patterns of AOD with period averages of 0.6 and 0.7, respectively (Fig. 4a). However, WRF-Chem underestimated the observed peaks mainly on 4th and 18th August 2006. The model underestimation could be associated with the calculation of the threshold wind speed because the magnitude of dust emissions to the atmosphere depends on the surface wind speed and soil features. The simulated SSA values show an agreement with the observed ones from ARM with period averages of 0.86 and 0.84, respectively (Fig. 4b). A similar study during 6–10 January 2006 showed that the simulated SSA ranges between 0.86 and 0.94 since the mineral dust complex index is very uncertain^45^.

### Diurnal cycles of radiation fluxes

**Figure 5:**
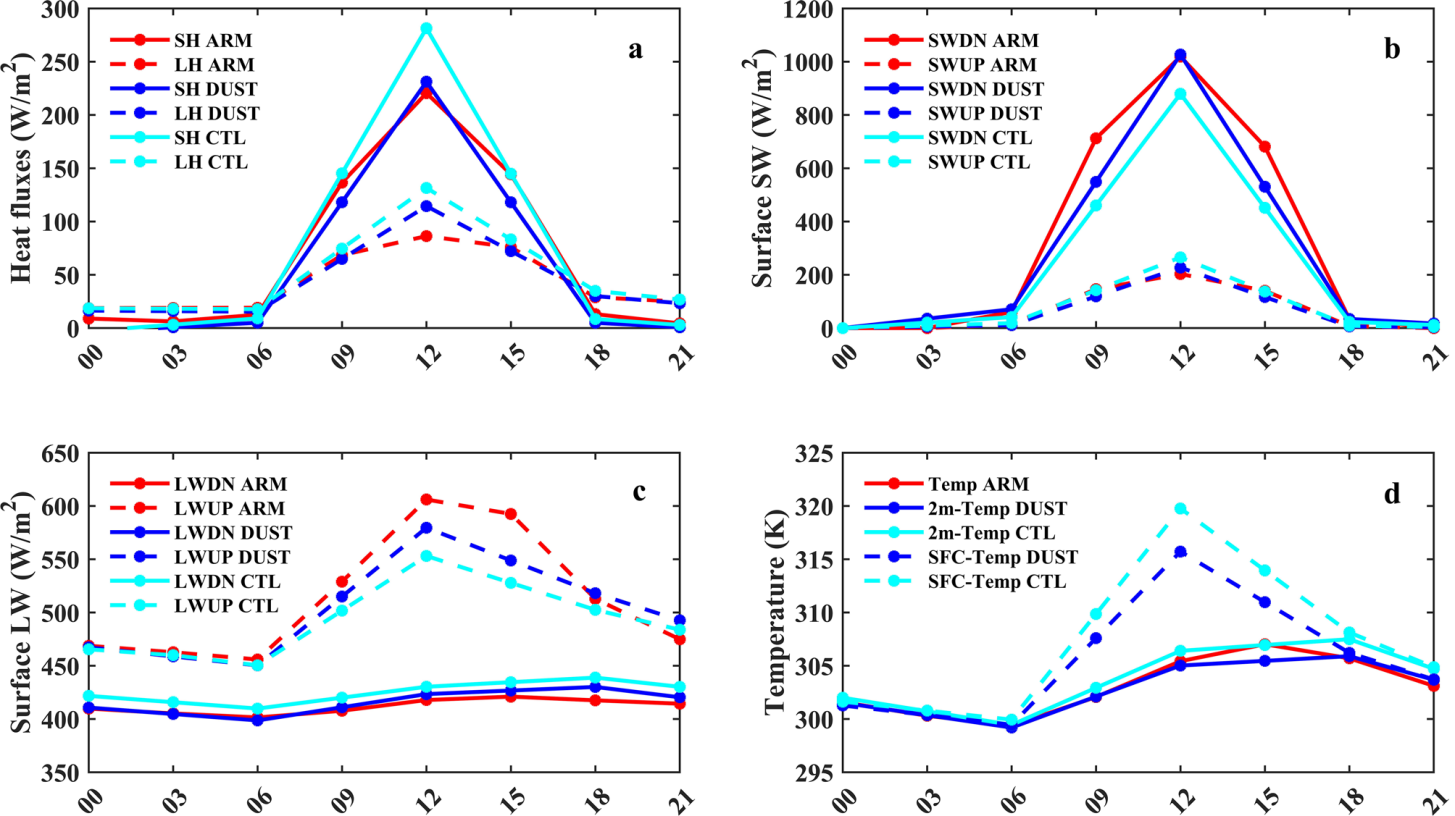
Mean diurnal cycle of SFC fluxes (in W/m^2^) and temperature (K) from ARM Facility Mobile observation at the Niamey site (13 N, 2E) and WRF-Chem simulations with and without dust over June–July–August 2006. Results are shown for: (**a**) SH, LH. (**b**) SWDN, SWUP. (**c**) LWDN, LWUP. (**d**) SFC Temperature, 2m-Temperature. The ARM station in Niamey is indicated in Fig. 1 in red color.

A frequently faced challenge in assessing the simulated diurnal cycles of surface energy fluxes is the lack of observations made from the surface to which models can be compared^107^. Figure 5 presents the evaluation of diurnal cycles of radiation fluxes and temperature between WRF-Chem and ARM observations at the Niamey site (13° N, 2° E). Table 2 summarizes the estimated metrics between ARM and WRF-Chem. The mean diurnal cycle in the SFC SH and LH heating is presented in Fig. 5a. The GH flux is not available from ARM observations at the Niamey site. The results show that simulated SH and LH agree well with observations from ARM, indicating similar variation with a peak at noon. However, results from dust simulation perform better with observations. For SH fluxes, normalized biases of 13% and − 8% and normalized root mean square errors of 20% and 33% were calculated for both dust and CTL simulations, respectively. For LH fluxes, normalized biases of − 3% and − 19% and normalized root mean square errors of 24% and 39% were reported for both dust and CTL simulations, respectively. Similar results were reported in Fig. 5b for the SFC SW spectrum radiations. The simulated SW downwelling and upwelling show better agreement with observations when dust aerosol in the model is considered. The reported metrics values between model and observations were normalized biases of 10% and 25%, and normalized root mean square errors of 25% and 42% for both dust and CTL simulations of SW downwelling spectrum radiation, respectively. In the SW upwelling spectrum radiation, normalized biases of 4% and − 14% and normalized root mean square errors of 24% and 35% were calculated for both dust and CTL simulations, respectively.

**Table 2:** Estimation of the model's ability to reproduce ARM observations using the normalized bias and normalized root mean square error.

	Normalized bias (%)	Normalized root mean square error (%)
**ARM sensible heat**		
DUST	13	20
CTL	− 8	33
**ARM latent heat**		
DUST	− 3	24
CTL	− 19	39
**ARM SW down**		
DUST	10	25
CTL	25	42
**ARM SW up**		
DUST	4	24
CTL	− 14	35
**ARM LW down**		
DUST	− 1	1
CTL	− 3	3
**ARM LW up**		
DUST	2	4
CTL	4	6
**ARM temperature**		
2-m DUST	0.05	0.2
2-m CTL	− 0.25	0.3
SFC DUST	− 0.8	1.4
SFC CTL	− 1.4	2.1

In the same way, as for SW spectrum radiations, LW spectrum radiations also were better simulated in the dust simulation compared to observations, as indicated in Fig. 5c. A satisfactory agreement between observations and model were reported for both dust and CTL simulations of LW downwelling and upwelling spectrum radiations. The estimated normalized bias and normalized root mean square error were of − 1% and 1% in the LW downwelling spectrum radiation for both dust and CTL simulations, respectively. Meanwhile, a normalized bias and root mean square error of − 3% and 3% were also estimated in the LW upwelling spectrum radiation for both dust and CTL simulations, respectively. Similarly, the SFC and 2-m temperatures were also well simulated compared to the observation made by ARM shown in Fig. 5d. However, the 2-m temperature better performs with ARM observation because ARM temperature is measured at 3 m. For the 2-m temperature, a better agreement was found from dust simulation given a normalized bias of 0.05%, and normalized root mean square error of 0.2%. In the CTL simulation, a normalized bias of − 0.25% and normalized root mean square error of 0.3% were recorded. Meanwhile, for the SFC temperature, normalized biases of − 0.8% and − 1.4% and root mean square errors of 1.4% and 2.1% were recorded in the dust and CTL simulations, respectively. The model performs well in simulating diurnal cycles of radiative fluxes and temperature, and very close to ARM observations in Niamey-Niger.

### Spatial distribution of radiation fluxes

**Figure 6:**
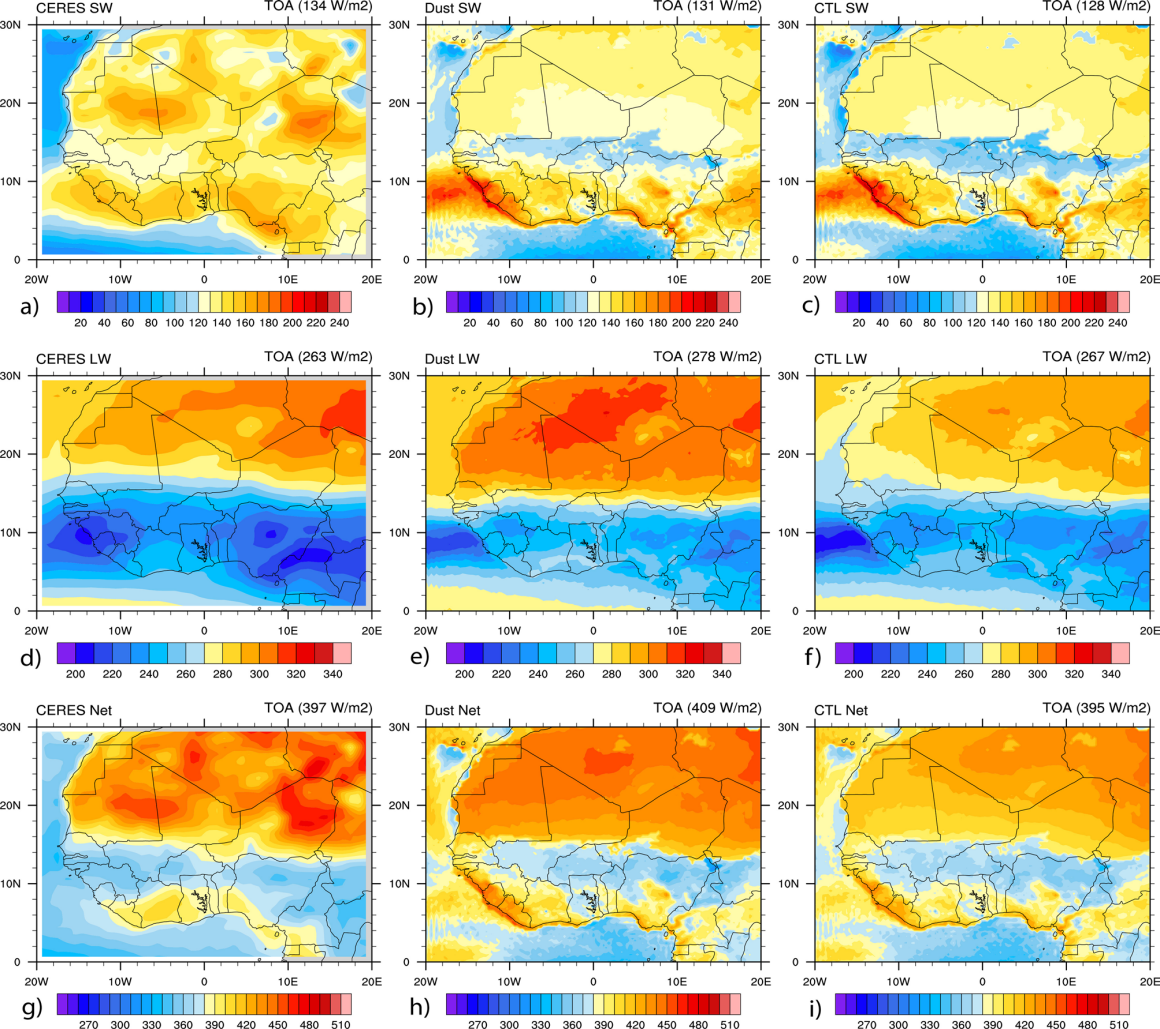
Spatial distributions of radiation fluxes at the TOA (in W/m^2^) from CERES and WRF-Chem with and without dust averaged over June–July–August 2006. Results are shown for: (**a**) SW from CERES, (**b**) SW from WRF-Chem dust, (**c**) SW from WRF-Chem CTL, (**d**) LW from CERES, (**e**) LW from WRF-Chem dust, (**f**) SW + LW from WRF-Chem CTL, (**g**) SW + LW from CERES, (**h**) SW + LW from WRF-Chem dust, (**i**) SW + LW from WRF-Chem CTL.

Figure 6 shows the comparison results of spatial distributions of SW, LW, and net (SW + LW) spectrum radiation at the TOA between CERES observations and WRF-Chem simulations averaged over the study period. Table 3 presents the mean values calculated over the study domain. Results reveal that all the simulated spectrum radiations (SW, LW, and net) show a similar pattern when compared to observations with maximum values over the desert regions (between 15° N and 30° N). The mean values of SW spectrum radiation over the study domain were estimated to be 134 W/m^2^ for CERES (Fig. 6a), 131 W/m^2^ in the dust simulation (Fig. 6b), and 128 W/m^2^ in the CTL simulation (Fig. 6c). In the LW spectrum radiation, the mean values reported were estimated to be 263 W/m^2^ for CERES (Fig. 6d), 278 W/m^2^ in the dust simulation (Fig. 6e), and 267 W/m^2^ in the CTL simulation (Fig. 6f). The mean values in the net spectrum radiations were calculated to be 397 W/m^2^ for CERES (Fig. 6g), 409 W/m^2^ in the dust simulation (Fig. 6h), and 395 W/m^2^ in the CTL simulation (Fig. 6i). The SW spectrum radiation was better simulated in the dust simulation in terms of both area mean and spatial configuration. But LW and net spectrum radiations were better reproduced in dust simulations in terms of spatial pattern while in CTL simulations in terms of the area mean nearer to ARM observations.

**Table 3:** Radiation fluxes at the TOA (W/m^2^) from CERES and CTL and dust simulations averaged over June–July–August 2006.

	TOA		
	SW	LW	SW + LW
CERES	134	263	397
DUST	131	278	409
CTL	128	267	395

**Figure 7:**
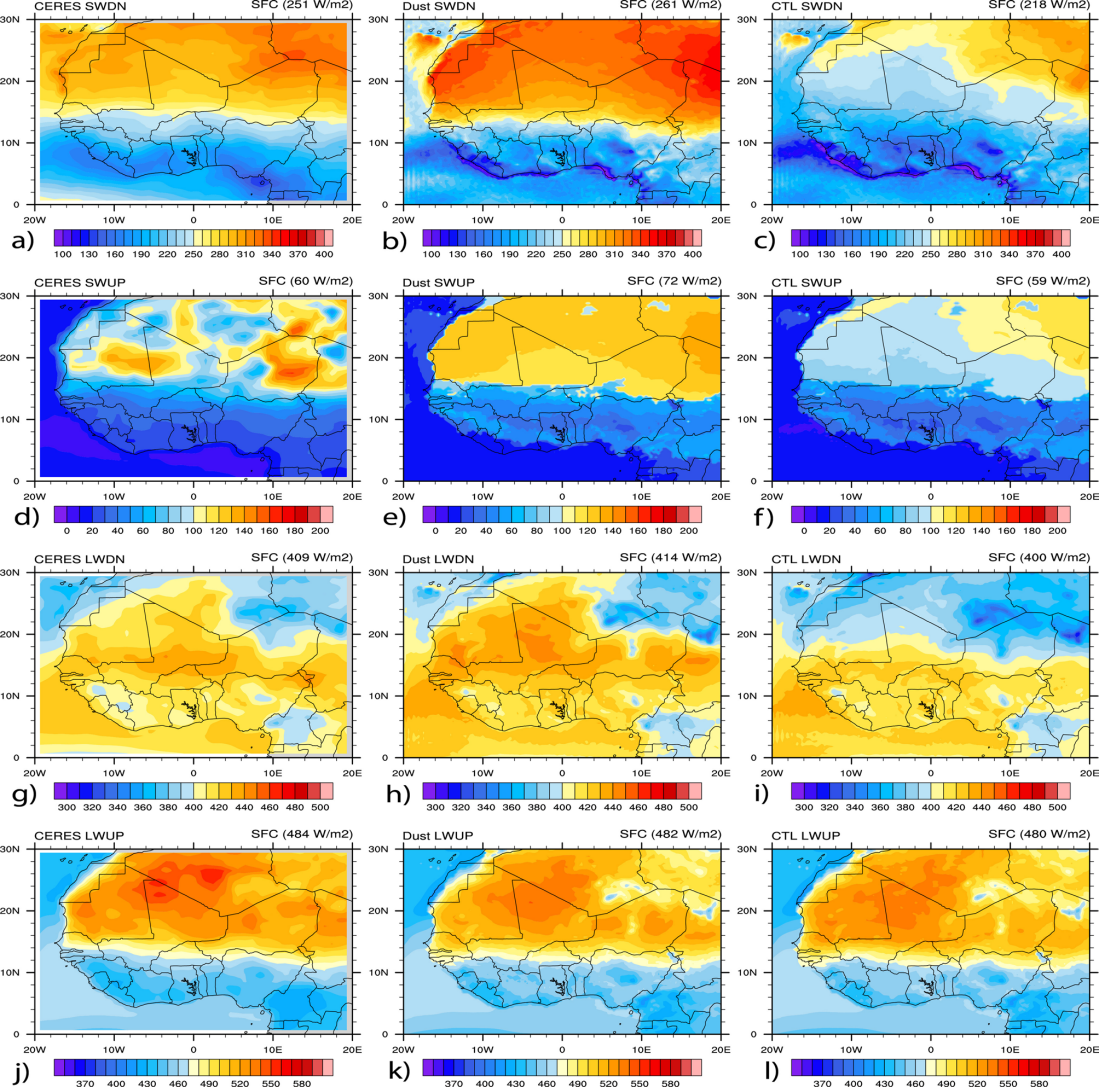
Spatial distributions of radiation fluxes at the SFC (in W/m^2^) from CERES and WRF-Chem with and without dust averaged over June–July–August 2006. Results are shown for: (**a**) SW downwelling from CERES, (**b**) SW downwelling from WRF-Chem dust, (**c**) SW downwelling from WRF-Chem CTL, (**d**) SW upwelling from CERES, (**e**) SW upwelling from WRF-Chem dust, (**f**) SW upwelling from WRF-Chem CTL, (**g**) LW downwelling from CERES, (**h**) LW downwelling from WRF-Chem dust, (**i**) LW downwelling from WRF-Chem CTL, (**j**) LW upwelling from CERES, (**k**) LW upwelling from WRF-Chem dust, (**l**) LW upwelling from WRF-Chem CTL.

Figure 7, the same as Fig. 6 but for downwelling, upwelling, and net radiation fluxes at the surface. Table 4 contains the means calculated for all spectrum radiations. Similarly, the results show that the model better performs in dust simulations for all spectrum radiations in terms of spatial patterns and mean values, except in SW upwelling spectrum radiation, where the CTL simulation shows better in terms of the mean value. In the SW downwelling spectrum radiation, mean values were estimated to be 251 W/m^2^ for CERES (Fig. 7a), 261 W/m^2^ in the dust simulation (Fig. 7b), and 218 W/m^2^ in the CTL simulation (Fig. 7c). The reported mean values in the SW upwelling spectrum radiations were 60 W/m^2^ for CERES (Fig. 7d), 72 W/m^2^ in the dust simulation (Fig. 7e), and 59 W/m^2^ in the CTL simulation (Fig. 7f). In contrast, for the LW downwelling spectrum radiations, the mean values were estimated to be 409 W/m^2^ for CERES (Fig. 7g), 414 W/m^2^ in the dust simulation (Fig. 7h), and 400 W/m^2^ in the CTL simulation (Fig. 7i). In the LW upwelling spectrum radiations, the mean values were reported to be 484 W/m^2^ for CERES (Fig. 7j), 482 W/m^2^ in the dust simulation (Fig. 7k), and 480 W/m^2^ in the CTL simulation (Fig. 7l).

**Table 4:** Radiation fluxes at the SFC (W/m^2^) from CERES and CTL and dust simulations averaged over June–July–August 2006.

	SFC		
	CERES	DUST	CTL
SWDN	251	261	218
SWUP	60	72	59
LWDN	409	414	400
LWUP	484	482	480
NETSW	191	189	160
NETLW	− 75	− 82	− 66
NET(SW + LW)	116	107	94

**Figure 8:**
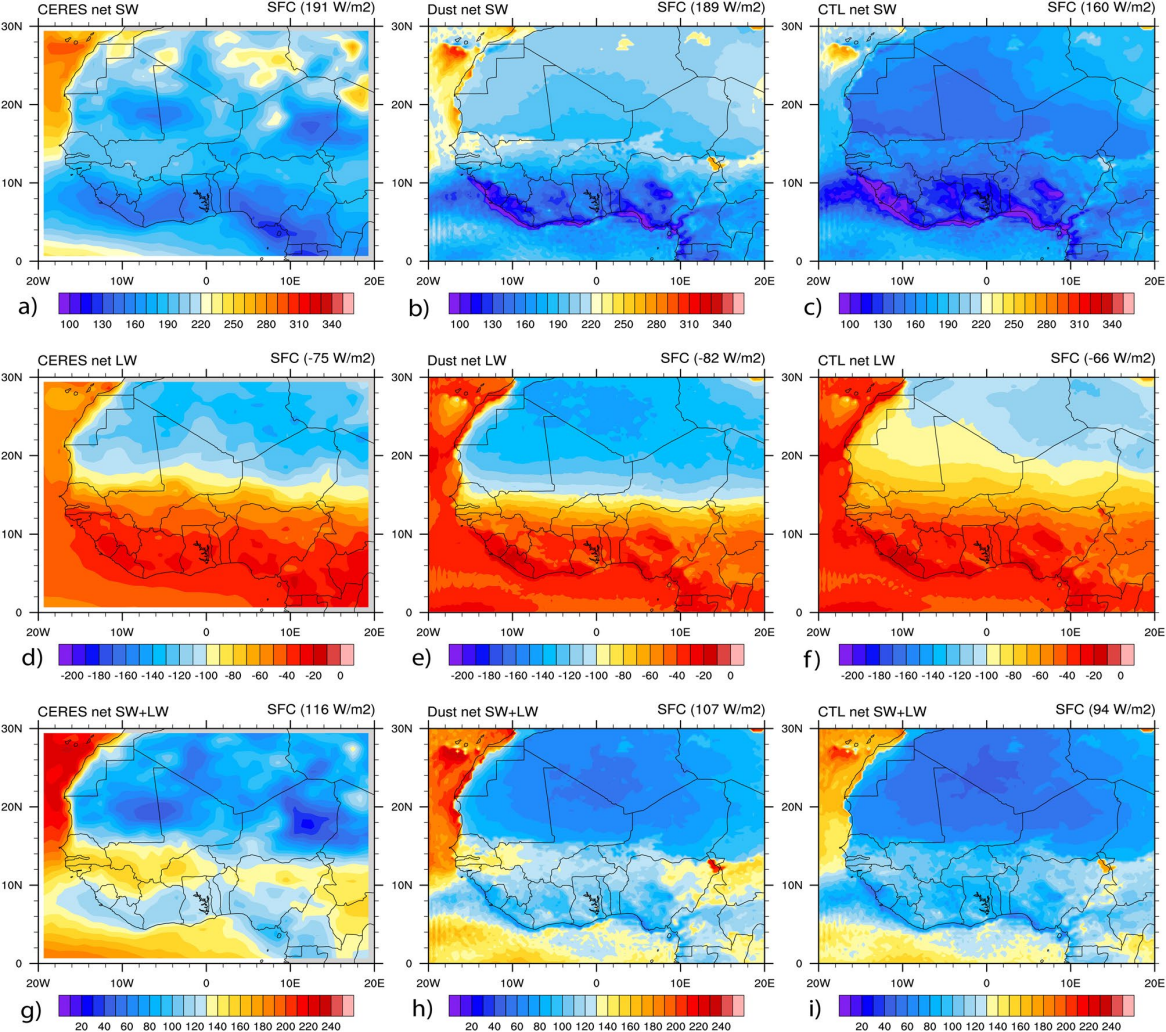
Spatial distribution of Net radiation fluxes at the SFC (in W/m^2^) from CERES and WRF-Chem with and without dust averaged over June–July–August 2006. Results are shown for: (**a**) net SW from CERES, (**b**) net SW from WRF-Chem dust, (**c**) net SW from WRF-Chem CTL, (**d**) net LW from CERES, (**e**) net LW from WRF-Chem dust, (**f**) net (SW + LW) from WRF-Chem CTL, (**g**) net (SW + LW ) from CERES, (**h**) net (SW + LW) from WRF-Chem dust, (**i**) net (SW + LW) from WRF-Chem CTL.

Figure 8 the same as Fig. 6 but for the net spectrum radiations (net SW, net LW, and net (SW + LW)) at the SFC. The results highlight that the model performs well in simulating the net spectrum radiations compared to CERES observations. Therefore, the dust simulation presents better agreement with observations than the CTL simulation in terms of spatial patterns and area means. The mean values over the study domain in the net SW spectrum radiation were recorded to be 191 W/m^2^ for CERES (Fig. 8a), 189 W/m^2^ in the dust simulation (Fig. 8b), and 160 W/m^2^ in CTL the simulation (Fig. 8c). In the net LW spectrum radiation the mean values were reported to be − 75 W/m^2^ for CERES (Fig. 8d), − 82 W/m^2^ in the dust simulation (Fig. 8e) and − 66 W/m^2^ in the CTL simulation (Fig. 8f). Similarly, in the net (SW + LW) spectrum radiation the agreement between model and CERES for the mean values were estimated to be 116 W/m^2^ for CERES (Fig. 8g), 107 W/m^2^ in the dust simulation (Fig. 8h) and 94 W/m^2^ in the CTL simulation (Fig. 8i). The WRF-Chem model performs very well with CERES observations over WA.

### Dust radiative forcing

**Figure 9:**
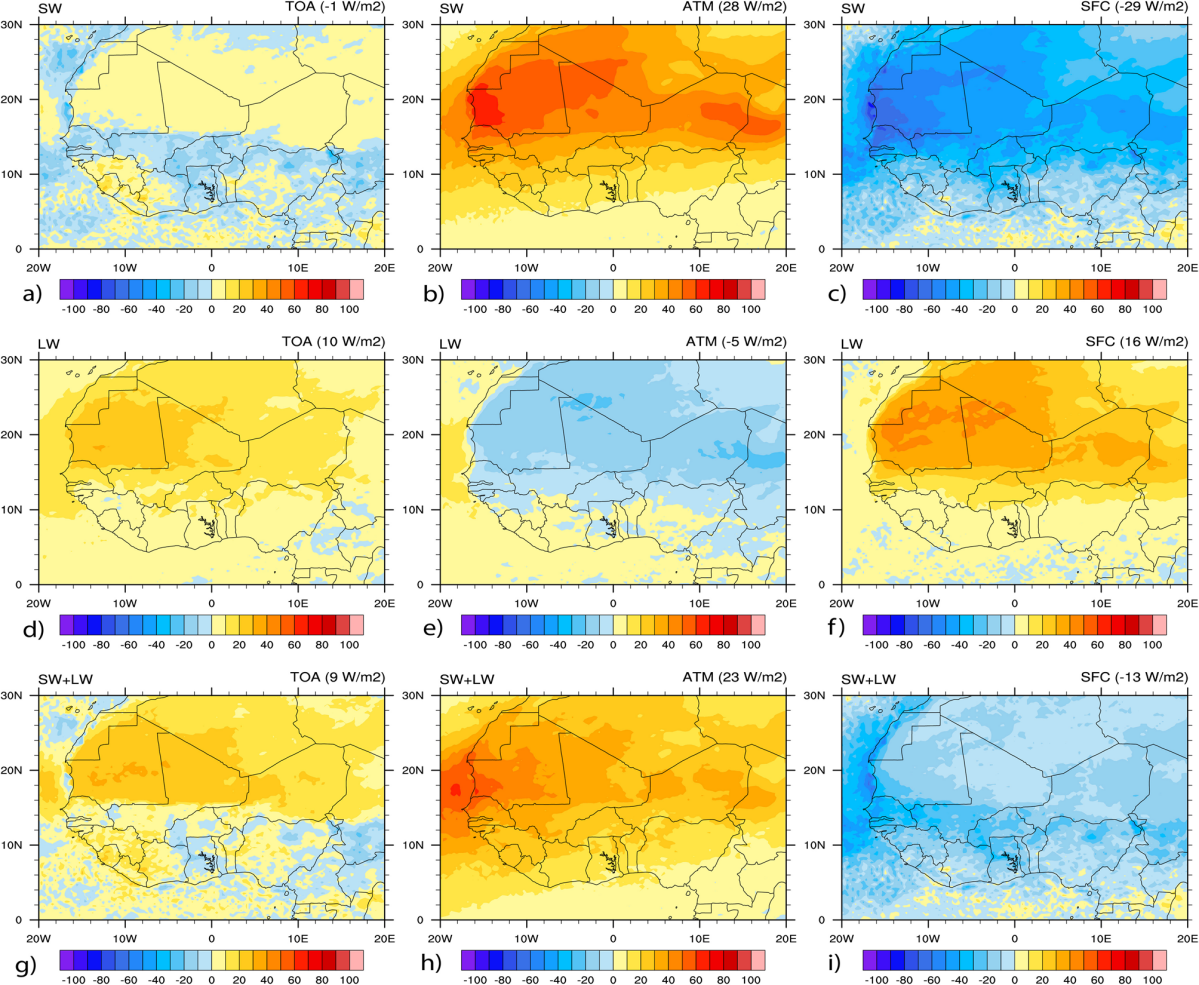
Spatial distribution of DRF (in W/m^2^) from WRF-Chem averaged over June–July–August 2006. Results are shown for: (**a**) SW at the TOA, (**b**) SW in the ATM, (**c**) SW at the SFC, (**d**) LW at the TOA, (**e**) LW in the ATM, (**f**) LW at the SFC, (**g**) net (SW + LW) at the TOA, (**h**) net (SW + LW) in the ATM, (**i**) net (SW + LW) at the SFC.

**Figure 10:**
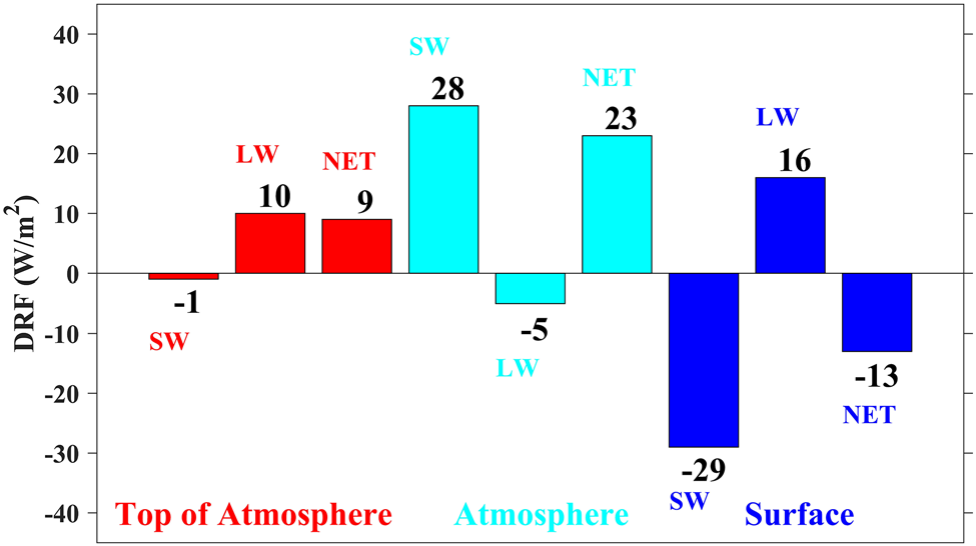
Mean DRF (in W/m^2^) from WRF-Chem over June–July–August 2006. Results are shown at the TOA in red color, in the ATM in cyan color, and at the SFC in blue color.

DRF remains poorly quantified due to both the low station's density over WA and poor knowledge of the spatiotemporal variability and properties of dust aerosols^89^*.* Studies have shown that dust aerosols have a highly distinct radiative forcing, and the effect sign depends on the surface properties, particle size distribution and aerosol composition that determine the single scattering albedo of the particles^108,109^. A positive radiative forcing tends to warm up the atmosphere, whereas negative forcing tends to cool it. Figure 9 presents the spatial distribution of DRF at the TOA, in the ATM and at the SFC averaged over the study period. Figure 10, same as Fig. 9, but for the estimated mean values bar plot over the study domain. Table 5 summarizes the mean values of DRF calculated at the SFC, in the ATM, and the TOA over WA during the study period. These highlights that the impact of dust on SW radiation reduces downward flux at the TOA (− 1 W/m^2^) (Fig. 9a), at the SFC (− 29 W/m^2^) (Fig. 9c), and increases absorption within the ATM (28 W/m^2^) (Fig. 9b). The reduction in downward flux is due to the upward reflection of SW radiation by dust particles in the ATM, particularly over the bright surfaces such as deserts. The absorption in the ATM is effective because the SW radiation reflected by the glossy surfaces is very likely to be absorbed by the dust particles^41^.

**Table 5:** Mean values of DRF (W/m^2^) at the SFC, in the ATM and at the TOA over June–July–August 2006.

	TOA	ATM	SFC
SW	− 1	28	− 29
LW	10	− 5	16
SW + LW	9	23	− 13

On the other hand, the LW radiation shows an opposite effect by increasing the absorption of the incident flux downwards at the TOA (10 W/m^2^) (Fig. 9d) and SFC (16 W/m^2^) (Fig. 9f), which in turn reduces it in the ATM (− 5 W/m^2^) (Fig. 9e). This highlights the ability of dust particles to absorb LW radiation, which depends on the particle size^109^. The hot desert emits infrared radiation, and this infrared radiation interacts much more with larger particles that have shorter lifetimes and can travel shorter distances. In contrast, SW radiation interacts much more with small particles, whose lifetimes are more than five days (~ 1 week)^41^. Previous studies on direct and semi-direct effects of aerosols over North Africa have reported similar results of SW DRF. The first study found that dust aerosol reduces the downwelling SW radiation at the SFC by up to 58 W/m^2^ with an average of 22 W/m^2^ over North Africa. The second one reported a mean DRF of − 19.7 W/m^2^ at the SFC over northern Africa due to the absorption and scattering of the incident radiation.

Results show that the net DRF at the TOA is either negative or positive (see Fig. 9g). Accordingly, the TOA DRF is positive over highly reflective surfaces such as the Sahara desert (about 15° N–30° N, Fig. 9g). Similar results were reported by previous studies^41–43,45,110^ and can be explained by surface albedo values on desert regions reducing DRF due to SW radiation. Besides, a large amount of AOD in the source regions contributes to the maximum absorption/emission of the LW radiation and induces warming at the TOA. On the other hand, the net DRF at the TOA is negative below 15°N over the dark zones, i.e., the Gulf of Guinea and the vegetated lands (Fig. 9g). The sign change around 15° N of the TOA radiative forcing is due to the change in albedo values in the northern desert and the southern Sudanian savannah zone. It is also due to the reduction in the amount of dust away from source regions. The mean net value of DRF at the TOA was estimated to be 9 W/m^2^ indicated in Fig. 10.

In the ATM (Fig. 9h), the net DRF has positive values over the study domain, which shows the absorption of radiative energy by the atmosphere. Because of their absorbent properties, it is clear that dust aerosols produce a warming effect within the ATM. The mean net DRF within the ATM was estimated to be 23 W/m^2^ over the study domain (Fig. 10). At the SFC (Fig. 9i), the net DRF is negative and was estimated to be − 13 W/m^2^ shown in bar plot values in Fig. 10, which indicates a strong cooling at the SFC by dust aerosols. The SFC cooling is attributed to a reduction of SW radiation absorbed and scattered by dust particles. These are consistent with similar previous studies^32,49,44,45,^, which found that DRF leads to a strong cooling effect at the SFC over Africa north of the equator. The net DRF at TOA is smaller than in the ATM or at the SFC over WA, illustrated in Fig. 10.

### Anomalies in surface heat fluxes, and temperature

**Figure 11:**
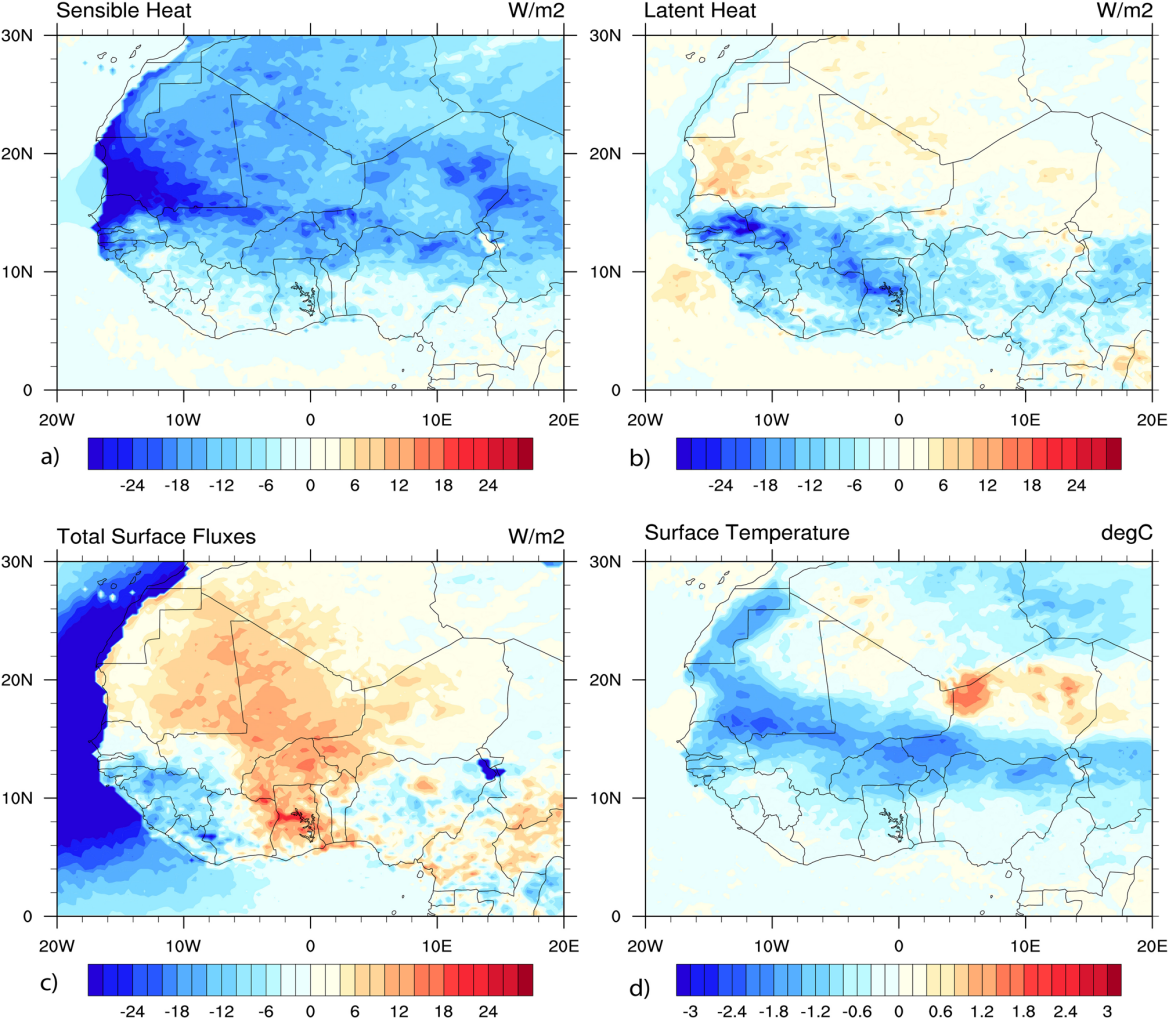
Changes in simulated SFC fluxes and temperature, induced by dust from WRF-Chem averaged over June–July–August 2006. Results are shown for: (**a**) SH, (**b**) LH, (**c**) Q, (**d**) SFC temperature. All fluxes use upward-positive convention, except Q, which is positive downward.

To maintain radiative equilibrium, the Earth must balance as the incoming solar radiation, and outward infrared radiation have to be equal^39,56^. Figure 11 investigates the impact of dust aerosols on the surface energy fluxes over the study period. The effect of dust induces negative anomalies of SH flux (positive upward) (up to − 24 W/m^2^) over the land surface, mainly over the WA monsoon region (Fig. 11a). Studies using model and field data have also emphasized a substantial reduction of SH flux due to dust aerosols^36,44,56^. The decrease in SH flux can be associated with a reduction of SFC temperature and precipitation, although radiative warming within the aerosol layer may induce a local increase in rainfall^43,56^.

Conversely, dust was found to cause positive anomalies of LH flux (positive upward) (Fig. 11b) above 15° N over the Sahara desert (up to 12 W/m^2^). These positive anomalies of LH flux lead to a loss of surface energy by evaporation. Over the dark green part, negative anomalies of LH flux (energy gain) were found below 15° N due to dust aerosols (up to − 24 W/m^2^). For the surface energy balance (Q, positive downward) (Fig. 11c), dust also causes positive anomalies (energy excess) over the land (up to 12 W/m^2^). Similar results were reported by studies using field data, regional and global climate models. They found that Q over WA is significantly altered and responds to variation of aerosols in atmospheric composition, clouds and water vapor^36,44,56^.

Similarly, Fig. 11d examined the impact of dust on the SFC temperature over the study period. Dust aerosols induce negative anomalies of SFC temperature over countries bordering the Atlantic Ocean, the Gulf of Guinea, over the Sahel, Western Sahara, Libya, and northeastern Algeria. Many studies have reported these negative anomalies and could be related to a reduction of solar radiation reaching the SFC over WA region^42–44,48^. However, there are regions with positive anomalies causing a warming effect over the SHL region and above the desert of Niger associated with a strengthening of the greenhouse effect by dust particles. Previous reports found these positive anomalies between 20° N and 30° N while they appear above 30° N for the other^44,111^. These results highlight an agreement between WRF-Chem and other previous studies that used field data and climate models to investigate the impact of dust aerosols over WA.

## Conclusions

Dust Radiative Forcing (DRF) and its impact on the surface energy budget during summer 2006 are examined using the WRF-Chem model in this study. The performance of the model to reproduce AOD, SSA, radiation fluxes and the temperature was firstly assessed with MISR, CERES and in-situ data from ARM mobile facility at Niamey over West Africa (WA). The impact of dust on the surface energy fluxes and temperature was then investigated. Results show that WRF-Chem performs well with observations in reproducing the distribution of AOD, SSA, radiation fluxes, and temperatures. However, significant biases exist in the spatial distribution of AOD over the southern part of WA, which is possibly attributed to model configurations, particularly the calculation of threshold wind speed in the scheme, dust emission scheme, boundary conditions, and surface properties.

It is found that the presence of dust particles induces a net warming effect at the TOA and in the ATM, and cooling at the SFC. The mean net DRF at the TOA is estimated to be 9 W/m^2^. Positive values of DRF are found over the reflective surfaces such as the Sahara desert. While over dark areas, such as the Gulf of Guinea and vegetated land, the effect is found to be negative. In the ATM, the mean net DRF is estimated to be 23 W/m^2^ indicating strong absorption of radiative energy by dust aerosols. At the SFC, the mean net DRF is estimated to be − 13 W/m^2^, which indicates a strong cooling at the SFC caused by dust aerosols.

Results also indicated that the SFC effect due to the presence of dust aerosols induces a significant reduction in both SH flux up to 24 W/m^2^ and SFC temperature up to 2 °C cooling effect over WA. Additionally, dust aerosols cause an increase of LH flux up to 12 W/m^2^ over the land above 15° N and a reduction up to 24 W/m^2^ over the dark green part below 15° N. This study highlights that dust aerosols significantly influence the surface energy budget over WA. Dust effects should be taken into account in further climate studies, to improve the accuracy in predicting weather and climate change. However, dust parameterizations still need to be improved. The study results provide supportive evidence of the potential value of this model for dust studies. The model can be used in a range of regional applications and will help to address key challenges, such as lack of ground observations.

## Data Availability

Data will be provided and made available upon the request of the readers.
